# Effects of Danazol on Clinical Improvement of Patients with Human T-cell Lymphotropic Virus Type I Associated Myelopathy/Tropical Spastic Paraparesis (HAM/TSP): A Placebo-Controlled Clinical Trial

**Published:** 2013-03

**Authors:** Reza Boostani, Hamidreza Saber, Mohammadmahdi Etemadi

**Affiliations:** 1 Department of Neurology, Mashhad University of Medical Sciences, Mashhad, Iran; 2 General Practitioners, Mashhad University of Medical Sciences, Mashhad, Iran

**Keywords:** Danazol, HTLV-I, Myelopathy

## Abstract

***Objective(s):*** Human T-Cell Lymphotropic Virus Type I (HTLV-I) associated myelopathy/tropical spastic paraparesis (HAM/TSP) is an endemic disease observed in Japan, Africa, Caribbean basin, and north-east Iran. It is usually presented as a chronic and progressive spastic paraparesis. There are some options for treatment of HAM/TSP patients. The aim of this study was to compare the effects of danazol controlled with placebo in relieving the symptoms and signs of HAM/TSP patients.

***Materials and Methods:*** Among 77 patients with definite diagnosis of HAM/TSP based on clinical and para-clinical findings, 71 patients had the required criteria for entering the study. Severity of symptoms and the degree of motor disability were determined before the beginning of treatment based on motor disability grading (MDG) in both groups of patients and were followed during 6 months in 1 month intervals for changes in symptoms and their motor disabilities.

***Results:*** Among 38 patients of the first group, after 6 months therapy with danazol, mean difference between MDG0 (before starting the treatment) and MDG6 (after six months), as an indicator of motor improvement in the patients, was 0.89. Meanwhile, among the 33 patients treated with identical appearing placebo, there was no significant difference between MDG0 and MDG6 (P< 0.001). Moreover, there was a significant difference in improvement of symptoms between two study groups.

***Conclusion:*** This study showed that danazol provides relative effects on improving motor disabilities and symptoms of HAM/TSP patients that can be considered according to its lower side effects compared to other suggested treatments such as corticosteroids, and its lower costs in particular patients.

## Introduction

HTLV-I is endemic in well-defined geographical regions throughout the world such as south of Japan, Sub-Saharan Africa, Caribbean countries and Brazil (1). In 1992, north of Khorasan (a province at north-east Iran) was concluded as an endemic area for HTLV-I infection. Most of the infected individuals remain asymptomatic, but it may cause various neurologic and non-neurologic presentations. Less than 5 percent of infected individuals will develop a chronic progressive paraparestic syndrome named HAM/TSP, a chronic inflammatory disease in central nervous system characterized by sphincter dysfunction, gait disturbances, spasticity, pain, and paresthesia (2). Other neurologic involvements such as neuropathy, cerebellar atrophy, myopathy, encephalopathy, meningitis, and cranial nerve paresis are rarely reported (3). No therapy has been shown to be completely effective on improvement of disease manifestations and progression. However, different studies have recommended various treatments mostly based on immunomodulatory strategies and down-regulation of pro-inflammatory cytokines, including corticosteroids, plasmapheresis, danazol, pentoxifylline, interferons, and some anti-viral agents such as zidovudine and lamivudine (4, 5). Nevertheless, many problems such as considerable side effects of corticosteroids and expensive costs of interferons limit their administration in many patients.

In this study, regarding the endemic infection of HTLV-I in north-east Iran and high prevalence of HAM/TSP patients and its associated disabilities in the region, we decided to investigate the therapeutic effects of danazol on these patients. Previous studies have shown some clinical improvements using danazol, but the main problems with most of these studies were the limited number of cases, short duration of treatment, and absence of control group (4,6). Therefore, we performed a placebo-controlled clinical trial on 71 patients with definite HAM/TSP diagnosis and examined the effects of danazol compared to placebo effects after 6 months. It should be noted that for a long time, danazol has been used as a treatment in endometriosis, and has well-known side effects; hence prescribing danazol does not have any interference with ethical issues.

## Materials and Methods

Before starting the treatment, HTLV-I associated myelopathy was proved in patients. To do so, all patients referred to neurology clinic of Ghaem Medical Centre in Mashhad, Iran with spastic paraparesia were screened using ELISA test for HTLV-I infection that was positive in 79 patients. In the next step, western blot (WB) test was performed, the result of which showed that among 79 patients, there were 77 positive, 1 negative, and 1 indeterminate. Then, polymerase chain reaction (PCR) test was performed to find HTLV-I genome in peripheral white blood cells on all patients. It was positive in 77 patients that had already positive WB and ELISA test results, and was negative in patients with negative and indeterminate WB results. For ruling out the other causes of spastic paraparesia, MRI of the brain, and cervical and thoracic regions of spinal cord were performed in all patients, and their CSF samples were studied for inflammatory patterns and Anti-HTLV-I anti-body. Apart from definitive diagnosis of HAM/TSP, the other criteria for entering the study included cases who : 1- had the age less than 65 and more than 20 years old; 2- were not pregnant or breast feeding, if females; 3- did not have any chronic disease like congestive heart failure, chronic renal failure, hypertension, and/or history of liver function abnormalities; 4- had cooperation for a six-month treatment period; and 5-had suspended other treatments for HAM/TSP, e.g. corticosteroids, at least four months before entering the study. So, the patients with definitive diagnosis of HAM/TSP who did not fulfil these criteria, or did not agree on taking CSF samples for diagnosis confirmation were excluded from the study. Side effects of the medications and other choices were explained to the patients and a written consent was obtained. The Local Research Ethics committee approved the study (80264). 

This was a placebo-controlled clinical trial, and the patients were divided into 2 study groups in a way that, except for the treatment, they were equal in other variables such as age, duration of symptoms, onset of the disease, severity of the disease, and sex ratio. In the first group, danazol was prescribed 400 milligrams per day (the initial dose was 200 mg per day which was increased to 400 mg during two weeks) for 38 patients, and in the second group, identically appearing placebo was prescribed for 33 patients, which were produced in the same form.

Both groups were carefully examined in the beginning of treatment during 6 months and in one-month intervals for monitoring the presence of side effects of the drug, effects of treatment based on motor disability grading (Table 1), and the value of patients’ satisfaction in improvement of their symptoms. The degree of motor disability was expressed based on MDG. MDG0 (the degree of motor disability before the beginning of the treatment) also indicated the severity of disease. The difference between MDG0 and MDG6 (the degree of motor disability after 6 months of treatment) represented the degree of improvement. Also, before and during the treatment, liver function tests were evaluated. 

Patients’ satisfaction in relieving symptoms (pain, paresthesia, urinary problems, and gait disturbances) was determined by the patient in percent. So, the degree of improvement was categorized in 5 groups: 1-no efficacy (no improvement), 2-mild efficacy (less than 25% improvement), 3-moderate efficacy (25-50% improvement), 4-good efficacy (50-75% improvement), and 5-excellent efficacy (75-100% improvement).

Data from clinical records were entered in SPSS (version 14) and checked twice, manually. MDG differences after 6 months were compared in 2 study groups with one-way ANOVA test. Drug effect on symptoms of patients (urinary problems, gait disturbances, muscle stiffness, pain, and paresthesia) in 2 groups was analyzed by chi-square test.

## Results

Among 77 patients with definitive HAM/TSP diagnosis, only 71 patients had the criteria for entering the study. Their mean motor disability grading (Table 2) before starting treatment (MDG0) was 4.2.

Mean MDG0-MDG6 (the difference between MDG before starting treatment and MDG after six months) in patients treated with danazol and placebo were 0.89 and near zero, respectively. Data analysis exhibited a significant difference in MDG improvement between the two groups (*P< *0.001). Also, there was a significant statistical difference in improvement of all symptoms including pain, muscle stiffness, urinary problems, and gait disturbances in patients treated with danazol compared to the second group who were treated with matching placebo (Table 1).

Among patients treated with danazol, 8 patients developed the following side effects: gastrointestinal disturbances such as nausea and epigastric pain in 2 patients, lower limb edema in 2 patients, hypertension in 1 patient, skin rash in 1 patient, mild increase of liver enzymes in 1 patient, and hirsutism in 1 patient.

## Discussion

According to the results, danazol has both positive subjective (reducing pain, muscle stiffness, urinary problems, and gait disturbances) and objective (based on clinical findings of MDG improvement) effects. Although it was not in the range of good and excellent improvement, these relative effects are noticeable according to the lower side effects compared to corticosteroids, and its lower costs compared to interferons. 

It is not precisely clear how danazol can improve signs and symptoms of HAM/TSP patients, but it probably affects the regulating immune system. In fact, after many years using danazol for treatment of endometriosis, its regulatory effects were found in certain immune processes. Danazol may induce a decline in serum immunoglobulins (7), level of serum C3 (8), and CA125 (9, 10). It may also increase the serum level of C4 (8), and inhibits Interlukine-1(IL-1) and Tumor Necrosis Factor (TNF) production (11). So, danazol is recommended in some immune system diseases like autoimmune hemolytic anemia (12), heredity angioedma (13), systemic lupus erythematosis (8), idiopathic thrombocytopenic purpura (14, 15), and HAM/TSP (4-6).

Some effects of danazol (like inhibition of IL-1) are dose-dependent, but the appropriate dose of danazol in HAM/TSP patients is not identified yet and this study did not answer to this question.

Data analysis showed no difference in the degree of drug effectiveness based on age (*P=* 0.22), sex (*P=* 0.94), severity (*P=* 0.62), and duration of disease (*P=* 0.73) among HAM/TSP patients. Also, the tests performed for evaluating danazol effects represented higher value in improving muscle stiffness (*P< *0.001) and lower value in improving paresthesia (*P=* 0.007). In other words, patients had less satisfaction for improving the paresthesia.

Side effects of danazol were seen in 8 patients. Fortunately, these side effects were resolved soon after stopping the medication. No life-threatening side effect was seen in our patients, which is similar to the other studies in which danazol +-had been used for treatment of endometriosis. For minimizing the side effects and increasing the tolerance of the drug, it is recommended to start with low doses followed by gradual increasing. In case of some irreversible complications like hirsutism and voice change, the medication should be stopped permanently and as soon as possible. 

## Conclusion

As a suggestion, danazol can be used for treatment of HAM/TSP patients. It is particularly recommended in patients who are not able to use corticosteroids or interferons because of their side effects and expensive costs. However, it should be considered that danazol has probably lower effectiveness than these medications. Its effect is not related to the severity of symptoms, or age and sex of the patients.

As mentioned above, this study does not demonstrate the most effective dose and duration of the treatment with danazol; hence further studies need to be conducted. 
